# The Preparation of the Mouth for and the Insertion of Artificial Teeth

**Published:** 1886-08

**Authors:** John Trude Fripp


AMERICAN JOURNAL

OF

DENTAL SCIENCE.

VOL. XX. THIRD SERIES.—AUGUST, 1886. NO. 4.

ARTICLE I.

### THE PREPARATION OF THE MOUTH FOR AND THE INSERTION OF ARTIFICIAL TEETH.

BY JOHN TRUDE FRIPP, L. D. S., EDIN. & I.

(Read before the Students' Society of the National Dental College.)

*Mr. President and Gentlemen:* The subject which I have chosen on which to invite discussion this evening is one which, in the view of some members of the profession, does not take very high rank in the scale of scientific subjects. There are some, I believe, who consider that the profession should be divided into two branches, one (the higher) the operative; the other (the lower) the mechanical.

I have heard, although I do not personally know it for a fact, that there are some practitioners who confine themselves entirely to operating, leaving the insertion of artificial teeth to other men.

Whatever may be the case in some comparatively few instances, there is no doubt that the vast majority of the men in our profession lay themselves out for general practice, or, to quote a phrase seen sometimes in advertisements, "Dentistry in all its branches."

Although it may seem very respectable and refined (especially from their own point of view) for a few men to speak of the operating room as the only sphere worthy of their presence and attention, yet the fact remains that, speaking generally, the man who is not a good mechanic will not be a good or successful dentist.

The powers that be, recognizing this fact, have in their wisdom required that the first part of the dental curriculum shall be a three years' apprenticeship to mechanical dentistry.

In no profession is the demand greater for versatility, for readiness of adaptation to any and every circumstance that may arise, than in our own. A man should be equally at home with the engine or the lathe, the forceps or the blowpipe, filling teeth or mounting them, conserving the mouth or supplying its deficiencies. The man who is not able to perform every detail of work, whether in surgery or workshop, for himself, will never be able, should he ever get the opportunity, to direct assistants in their work for him, or to decide whether the work is or is not properly performed. Such a man would be scarcely likely either to make or maintain a successful practice.

Be a man ever so good an operator, yet if he have not the tact or skill to make a good fit or a suitable match, his reputation is likely to be of very slow growth and his connection correspondingly small; while a poor or indifferent operator (that is, in the matter of saving teeth) will, in many cases, if he be clever and skilful in the adaptation of artificial teeth, rapidly make a large and remunerative practice.

Mind, I am not saying that this should be the case, but simply recognizing what I believe to be a well-known fact. I believe very thoroughly in conservative practice, but seeing that so great a demand exists for what the Americans call "Prosthetic Dentistry," a demand which is probably greater now than at any previous time, and which is constantly increasing, is there not room for very great improvement in this branch of our work, and should not greater and more intelligent care be bestowed upon it?

How many of the cases which come to us give the idea that the operator took the models and forthwith handed them to an assistant in the workshop, with few or no instructions, leaving him to "set up" the teeth according to the best of his ability, without any regard to the character of the mouth and face with which the new denture should harmonize? The case thus "mechanically" made is put into the mouth whether satisfactory or not, in a large number of instances decidedly *not*. Now this serious fault is committed not only by those from whom it might be expected, that is the advertising quacks, who may be supposed to make teeth by steam machinery, but many of those who hold good names and high places in the profession are guilty of inserting in the mouths of confiding patients work which I venture to say they would not care to meet again in the presence of their professional brethren.

The only excuse which can be urged is want of time, and this should be no excuse at all, since if a man have not time to do work properly himself or see that it is done properly by others he ought not to undertake it.

It is probable, however, that with most of us the danger of having too much work to do is a somewhat remote one, and if we do not our best to meet the requirements of each case entrusted to us we cannot shelter ourselves behind this flimsy excuse.

In my paper this evening I do not expect to advance anything new, and it will no doubt be found as the discussion proceeds that my own knowledge is sadly deficient in many respects. The fear of disclosing my own ignorance does not in any way deter me from introducing the subject, because my object is not to teach but to learn, and to elicit discussion which shall be mutually profitable. It is impossible for me to speak too highly of the advantage which I

have personally derived from the meetings of our Society, and I am hoping that this evening I shall go home with a larger store of knowledge than I brought with me. To proceed now to the direct consideration of my subject. "The preparation of the mouth for and the insertion of artificial teeth."

A large number of the patients who consult us for the purpose of getting supplied with artificial teeth (perhaps I ought to say the *larger number*) are those who have greatly or entirely neglected the mouth in the past, and have simply allowed it to go to ruin. As the consideration of such a case will afford us the best means of dealing with our subject, I will take a supposititious one which will find its counterpart in actual every-day practice.

A patient presents himself, with a mouth in which half of the upper teeth are irretrievably lost. Two or three front teeth too bad to make a decent appearance if filled, or decayed away to the stumps. Some molars and bicuspids on both sides gone also, stumps remaining. In the lower jaw six front teeth probably in good condition, that is free from decay, but much covered with tartar; perhaps one or both bicuspids either side also remaining: first molars have been extracted, second and third molar stumps still existing.

I think this is a fairly typical case. What shall we do with it?

The old plan, which I was taught, and which I regret to say, I practiced in my early days, was to get, first, the nippers and stump file, and proceed to reduce all the ragged edges to the level of the gum—not to extract anything unless the patient was in actual pain. Models taken, the pieces were made, materials according to the means of the patient, but always held in by three or four wires or bands in the upper, two clasps in the lower. Simple, expeditious mode of treatment, no pain given, patient pleased, fee paid, operator satisfied. The sequel of such a case is well known. Fairly comfortable for a time, but mouth never healthy, breath unpleasant, the denture being only seldom (in some cases I have known by the advice of the dentist, never) removed, the patient finds after awhile that the supporting teeth are becoming sensitive, and that any effort to remove the plate produces great pain and discomfort. Besides this injury to the supporting teeth, the stumps also are often tender; a slight cold, or other derangement of health, giving rise to periostitis and gingivitis, and making the toleration of the teeth for a time almost impossible. On raising the lips we shall probably find a number of more or less active volcanoes in the shape of disgusting pus-discharging sinuses. Either the dentist is again consulted and a little patching done, or the patient waits in constant uneasiness until all the remaining teeth are lost, and then the work has to be commenced all over again. Either all the roots must be removed, or more likely a complete set made over them, to be kept in place by springs, and the roots left to be thrown off as nature can best manage that process.

This is the plan which used to be adopted, and which, I am sorry to say, is not discarded yet by many who ought to know better.

I cannot too emphatically say that such a course is to be most strongly condemned, unless the patient's health or some other urgent and imperative reason stand in the way of proper treatment. It should then only be undertaken after explanation or under protest.

Discarding this "old way," as I am sure we shall all be agreed to do, again we ask, "What shall we do with the case?" A great authority, Mr. Spence Bate, has recently said, in a paper on "Excision versus Extraction," read before the British Dental Association, that "neither stump nor tooth should be removed that is healthily implanted in its alveolus or could be made so." And then proceeds to an enumeration of some of the cases in which roots should or must be removed, all the healthy ones being treated and filled. We have the greatest respect for this most thorough conservative surgery, and it must be a great pleasure to a man to be able to restore to perfect health a mouth full of stumps, without putting his patient to the pain of a somewhat formidable operation.

But, I would ask, how often does it come within the range of possibility, or at least of practicability, to treat and fill a large number of stumps? Supposing the operator to be willing, the patient would, I think, seldom be found who would be able or willing to give the required number of sittings, or pay an adequate fee for the time and labor employed.

Again, however good in theory, it seems to me to be bad in practice to remove here and there a diseased root and leave here and there a good or healthy one. The surface of the jaw is rendered very irregular, the yielding gum and the unyielding stumps alternating and making the comfortable fit of a denture much more difficult to obtain.

Of course where only upper front stumps are in question they should, if possible, be treated and pivoted. In such a case there could be, I think, but little difference of opinion.

There is yet another reason why, except for the purpose of pivoting or otherwise fixing artificial crowns, dead stumps should not be left in the mouth, and that is that they are so frequently exostosed, and give rise to more or less severe neuralgic pain and disturbance, the patient in many cases being entirely unaware of the cause of the pain, and in some cases I have known obstinately sceptical when the cause has been pointed out.

It will no doubt have already been gathered from the tone of my previous remarks what my own treatment would be of such a case as that instanced.

I should carefully look round the mouth, and a few minutes' inspection would enable me to decide what teeth should remain and what be removed. Having due regard to the position of the teeth and the antagonism of the jaws, all the sound teeth should be left, and also such teeth as can be made good by filling, and all tartar carefully removed.

*All doubtful teeth*—and on this I would lay great emphasis—should be extracted, and also all the stumps. Many an otherwise satisfactory case has been to a great extent spoiled by leaving in the mouth one or more teeth which (it was thought) might be made good, but which did not prove amenable to treatment, and eventually had to be extracted, much to the chagrin of the operator and dissatisfaction of the patient.

I know that several objections may be urged against this mode of treatment. Some will say that the shock to the patient in a case of such extensive extraction will be too great. As a matter of actual practice I have found that generally a patient suffers little if any more shock or after-pain from the removal of a dozen than from the removal of three or four teeth.

Another great objection frequently urged is that there is so much difficulty in the length of time the patient must wait for the settling of the gums; a period of several months or a year. I put in a set of teeth some time ago for a woman who had all her teeth extracted at a Dental Hospital, and, acting on the advice there given her, had waited in a perfectly edentulous condition for twelve months. I also saw in the *Dental Cosmos* some time ago an advertisement of a practice for sale in which one of the points urged was "so many dollars' worth of work in hand" in the shape of mouths waiting until fit for the insertion of artificial teeth.

To the practice of keeping the patient waiting thus without teeth there are most serious objections. It is almost like condemning him to a slow starvation, for little if any solid food can be taken. The muscles of the mouth and face become so seriously contracted that the natural expression is greatly interfered with and can never be properly restored, and in many cases the contraction is so great that a set of the proper size cannot possibly be inserted.

A year or two ago I heard a conversation in the extraction room below between several gentlemen, one or two of whom were officers of the hospital. The subject was the difficulty of making an equitable arrangement in the charges for temporary and permanent sets of teeth. If a temporary set were inserted the patient did not expect to pay the full fee, and yet for any subsequent set they objected to the full fee, saying, "Mr. So-and-so had only charged *so much* for a set."

This is a difficulty which in actual practice I do not find to exist. For some years past I have ceased to talk about temporary sets as I do not believe them necessary.

My plan for a long time past has been to put the new case in from twelve to twenty-four hours after the extraction, or even less, explaining to the patient that perhaps at the end of a year a refit will be necessarry; but as an actual fact it is very seldom required, the gums to a great extent shrinking and settling to the shape of the denture.

I will just instance a case which I had the opportunity of seeing during the last month. A lady from Yorkshire came to consult me about putting her mouth in order, and arranged to stay with her friends close by until I had finished. This was about two-and-a-half years ago. I administered the gas to her twice each day for three successive days and removed twenty-six teeth. Within a few days I put in a new set, vulcanite upper, cheoplastic lower, telling her she would probably want them remodelled in a year's time. I saw her once or twice to relieve the set during the next week or two, and she then returned to the North. I heard from her friends again and again that she was very comfortable, and now at the end of two-and-a-half years she assures me they are more comfortable than ever, and she can speak and eat with the most perfect comfort. I should have said she came to see me because a good man in York City had told her he must keep her without teeth for three months.

Since adopting this plan I bought Mr. Oakley Coles' book on "Dental Mechanics," and there found to my great satisfaction that I was in most excellent company. If you will allow me I will give you a paragraph from his book *in extenso.*

He says, "The question how soon after extraction artificial teeth may be inserted is one of great perplexity if the operator be unguided by practical experience. Theoretically, one would consider that a considerable time should be allowed to elapse. From my own experience, practically, I consider twenty-four hours enough; that is, I have many times taken out ten or more teeth one day, and put in a full set of artificial teeth the next day, and I have found the least absorption, especially in comparatively young subjects, in those cases where the shortest time has elapsed between the operation and the insertion of a new denture. Beyond the advantage of ready treatment which this plan offers, there is the still greater benefit of preserving more completely the contour of the face. Many practitioners consider that a temporary set of teeth may be fitted in at the end of a fortnight or three weeks, and a permanent set at the expiration of twelve or eighteen months. I have found, however, that those dentures that I have fitted in immediately after operating have fulfilled every requirement of a permanent set, so that no further change has been necessary."

This exactly coincides with my own experience, but I must add to it a little fuller explanation of my own mode of practice.

The impression being taken immediately after the extractions, with composition as soft as possible, and kept in the mouth a good while to get hard enough to be withdrawn without "dragging" or "sucking," it is put into cold water and sent to the workshop to be cast immediately. I then select the exact teeth for the case, and try them by putting them right up into the sockets, and I then know that those teeth will fit in without any grinding or fitting, going into the socket from an eighth to three-eighths of an inch. I am referring now specially to the six or eight front teeth, though in many cases the molars also may be put a considerable way into the sockets.

In a case which I have in hand while writing this paper I have removed all the back teeth on both sides in the upper first, leaving the six fronts until last, in order that the sockets may only be vacant one day.

When the model is cast it will be found that the sockets are well marked though not deep enough. Each socket must then be carefully deepened until the tooth will fit into the model as deeply as it did into the gum. When all the teeth are thus placed in position they may be waxed on to the model, and the palate being put in the piece will be ready for flasking and baking.

In cases where the bite is close, or where we desire to make a specially strong and good frame, flat teeth may be chosen and backed either with gold or dental alloy, and when fitted into their sockets a wire bent round so as to touch every tooth, the whole waxed together and carefully lifted off the model and invested in plaster and sand for soldering. By this plan an exceedingly light and strong case can be made.

In cases where only three or four teeth are required the plan I have described is exceedingly satisfactory. I will mention one which I did a week or two ago. I removed one central incisor and both anterior bicuspids at twelve o'clock, and at six put in the new teeth backed with dental alloy and strengthened with wire soldered to each tooth and the whole mounted on a vulcanite palate. Four days after, being on a visit to the patient's house, I examined the mouth, and found that on raising the lip it was impossible to tell which of the centrals was the new one, so perfectly had it fitted into the gum, and so exactly alike were the festoons of the gum over natural and artificial teeth.

One point it is very important to observe. The *teeth should always on first putting into the mouth be too long* by an eighth of an inch or so, as they sink into the gum considerably afterwards, say in the course of a day or two. Some cases which, when first put into the mouth, look as though indisposed to fit up in their places I find to work up into the gums and fit as snugly as could be wished.

Where only a few teeth have been extracted and the mouth is not very ragged, gold or dental alloy plates may be used if desirable and with perfect success. I have with me a couple of models in which gold and dental alloy were used, and you will see where the sockets have been cut to receive the teeth.

Of course there will be a subsequent shrinkage of the gum, and the festoon of which I have just spoken, will not in all cases be preserved ; but if the tooth be inserted far enough into the socket the shrinkage can seldom or never be sufficient to allow the end of the tooth to project or catch the lip.

We are all familiar with the appearance of the mouth in which teeth have been inserted a few weeks after the extraction. The teeth being short, the gums have gone away from them entirely, and the denture may easily be removed by the action of the lip upon the projecting and uncovered teeth.

In the plan I have been advocating the teeth are of necessity brought further in at the necks, indeed exactly in the position of those they supersede.

Another important feature is that the pain to the patient is much less when the teeth are put into the sockets.

When once the sockets are closed the teeth must be put outside the gum, and any pressure at once pinches the gum between the alveoli and the new denture, giving rise to great pain, and often necessitating a further shortening of the teeth at the cervical end.

I have found that after the first few hours or a day the pain and tenderness have in most cases almost entirely passed away. In order that the perfect adaptation may be maintained I usually instruct patients not to allow the new case to be out of the mouth at all (except for cleaning after meals) for some weeks, until the gums have fairly settled, and after that to use their own discretion about wearing it at night.

In cases where the lower back teeth have been extracted

I do sometimes think it better to wait for awhile, as the teeth cannot be put into the sockets as in the upper, but, as in the case I instanced just now, when it has been necessarry to insert at once, the teeth usually fit very well.

I have generally recommended a mouth wash of Condy's fluid or permanganate of potash, until the gums have well healed.

I should like to say here that, notwithstanding the very extraordinary case which Mr. Spence Bate mentions in the paper from which I have already quoted, of a woman who went mad after the extraction of a number of teeth, I believe that it is very rare for any untoward results to follow that operation. I cannot call to mind any other that I have heard of, although many times I have known patients to regret exceedingly that the stumps were *not* removed. I have never known a case during the nearly twenty years I have been connected with the profession of regret being expressed or any trouble ensuing after the removal. All my experience has been entirely the other way.

It will, no doubt, be felt by some that it must be somewhat inconvenient to be obliged to get the cases done so quickly, especially if several of them be in hand at once. It is inconvenient sometimes, but the result is worth the inconvenience and trouble. But if a judicious selection of teeth be made, there is so little fitting required that a case is very quickly got out of hand. I have in many instances cut the sockets in the model and put the teeeth all in order in ten minutes or a quarter of an hour from the taking out of the models, and given to my assistant to wax up and vulcanize at once.

Mentioning the fact of putting the teeth in order reminds me of one thing which I had almost forgotten to say, it is this—If men undertake to supply artificial teeth they should either see to the arrangement of them themselves or get an intelligent assistant who can see the patient's mouth and receive instructions in the surgery and then arrange the teeth artistically. How seldom do we see Nature's irregularities copied, and how often do we see, much to our disgust, rows of teeth "set up" as regularly as the keys of a piano and the edges as even as if smoothed with a file?

So frequently teeth are put into the mouths of elderly patients as perfect as we could wish to see at the age of blushing sixteen.

The depots provide us now with a marvellous assortment of teeth ready to hand, such as we can scarcely wish or imagine to be surpassed; but the hand and eye and brain of the dentist are all needed to so arrange and adjust them that they may be life-like, instead of obviously artificial. "The height of art is to conceal art" is a motto which every dentist should remember.

A little chipping of the corners, roughing slightly the polished surface, staining if necessary, and many other little devices which will occur to the mind in practice, go a long way in making our work satisfactory to ourselves and pleasing to our patients.—*London Dental Record.*

